# Anthocyanin Biosynthesis Induced by MYB Transcription Factors in Plants

**DOI:** 10.3390/ijms231911701

**Published:** 2022-10-02

**Authors:** Changxia Li, Wenjin Yu, Junrong Xu, Xuefang Lu, Yunzhi Liu

**Affiliations:** College of Agriculture, Guangxi University, Nanning 530004, China

**Keywords:** anthocyanins, MYB TFs, regulator pathway, target genes, interactions between proteins

## Abstract

Anthocyanins act as polyphenolic pigment that is ubiquitously found in plants. Anthocyanins play a role not only in health-promoting as an antioxidant, but also in protection against all kinds of abiotic and biotic stresses. Most recent studies have found that MYB transcription factors (MYB TFs) could positively or negatively regulate anthocyanin biosynthesis. Understanding the roles of MYB TFs is essential in elucidating how MYB TFs regulate the accumulation of anthocyanin. In the review, we summarized the signaling pathways medicated by MYB TFs during anthocyanin biosynthesis including jasmonic acid (JA) signaling pathway, cytokinins (CKs) signaling pathway, temperature-induced, light signal, 26S proteasome pathway, NAC TFs, and bHLH TFs. Moreover, structural and regulator genes induced by MYB TFs, target genes bound and activated or suppressed by MYB TFs, and crosstalk between MYB TFs and other proteins, were found to be vitally important in the regulation of anthocyanin biosynthesis. In this study, we focus on the recent knowledge concerning the regulator signaling and mechanism of MYB TFs on anthocyanin biosynthesis, covering the signaling pathway, genes expression, and target genes and protein expression.

## 1. Introduction

Anthocyanins act as natural water-soluble pigments which range from red to blue, existing as glycosides in combination with glucose or cellobiose molecules, which are widely distributed in the roots, leaves, flowers, fruits, and so on of many plants [1]. Anthocyanins are not only essential for plant performance, but also have beneficial effects on human health due to antioxidant, anti-mutagenic, and anti-carcinogenic capacities [2,3,4]. Therefore, how to improve the anthocyanins accumulation of plants has long been a focus of intense research.

The biosynthesis pathway of anthocyanins has been clearly clarified in plants [5]. Anthocyanin is biosynthesized by the phenylpropanoid pathway that is well known [5]. Briefly, the pathway starts with the condensation of one molecule of 4-coumaroyl-coenzyme A (CoA) and three molecules of malonyl-CoA, leading to naringenin chalcone [6]. This process is usually accompanied by CHS conduction, before the pathway diverges into side branches leading to different classes of flavonoids, including anthocyanins [7]. Anthocyanin accumulation is regulated by a series of structural genes [8]. Additionally, increasing studies indicate that many environmental factors affect anthocyanin biosynthesis during the past few years, including nutrient deficiency (nitrogen, phosphorus, sugars, magnesium, and CaCl_2_), light stress, UV-B irradiation, low temperature, wounding stress, the invasion of pathogenic bacteria, etc. [9,10,11,12,13,14,15]. Importantly, MYB transcription factors (TFs) are involved in the regulation of the anthocyanin biosynthesis pathway that has been identified. For example, in grapevine, VvMYBA1 and VvMYBA2 are identified that regulate the biosynthesis of anthocyanin [16]. In Arabidopsis, R2R3-MYB TFs including PAP1, PAP2, MYB113, and MYB114 as positive regulators are involved in the biosynthetic pathway of anthocyanin [17,18]. In apples, MdMYB1 has been shown to be responsible for controlling anthocyanin biosynthesis [19,20,21]. Continued research has shown that other TF families are involved in anthocyanin synthesis, including the MADS, NAC, HD-Zip, and ERF TFs, indicating that there are still many mechanisms of anthocyanin synthesis and regulation that are yet to be elucidated [22,23,24,25].

Most R2R3 MYB TFs involved in the control of anthocyanin biosynthesis are activators that enhance the expression of biosynthetic pathway genes, such as AtMYB75 (or PAP1), AtMYB90 (or PAP2), AtMYB113 and AtMYB114 in Arabidopsis [18,26], MdMYBA, MdMYB1, MdMYB10 and MdMYB110a in apple [21,27], SlMYB12 in tomato [28], and StAN1 in potato [29,30]. In addition to MYB activators, it has also been reported that MYB TFs can repress anthocyanin accumulation. There are two distinct classes of MYB TFs which negatively regulate anthocyanin accumulation: R3-MYB and R2R3-MYB repressors, which have one or two repeats of the MYB domain region, respectively. These R2R3 MYB repressors include AtMYB3/4/6 in Arabidopsis [31], MdMYB16/17/111 in apple [32], FaMYB1 in strawberry [33] and PhMYB4 and PhMYB17 in petunia [34]. The R3 MYB repressors have been reported in several plant species, including CAPRICE (CPC), TRIPTYCHON (TRY), and AtMYBL2 in Arabidopsis.

In view of the increasing interest in studies of MYB TFs in anthocyanin biosynthesis, this paper provides a critical review of the most recent work in the literature regarding various mechanisms associated with the anthocyanin biosynthesis of plants in which MYB TFs play a role. In addition, for genes/proteins, the expression of which is mediated by MYB TFs during the anthocyanin biosynthesis, are also briefly summarized. Therefore, this review may provide new ideas for future researchers studying the regulatory role of MYB TFs in the anthocyanin biosynthesis pathway. Additionally, it was an objective of this study to describe in detail the regulatory mechanisms that are relevant to the anthocyanin accumulation of plants in which MYB TFs are involved and further accelerate research into the use of MYB TFs in identifying new varieties and breeding new cultivars.

## 2. Effect of MYB TFs on the Anthocyanin Biosynthesis

### 2.1. MYB TFs Positively Regulate Anthocyanin Biosynthesis

It was reported that the MdMYB10 transcription factor had potential to enhance anthocyanin biosynthesis in apple fruit [21]; Table 1. Ban et al. [20] found that MdMYBA was responsible for controlling anthocyanin biosynthesis in apple skin. A novel MYB transcription factor was designated as MdMYB3 and found that it regulated the anthocyanin accumulation of apple fruits skin [35]. MYB110a, a paralog of MYB10, was responsible for red fruit-flesh phenotypes of apple [27]. Overexpression of MdMYB9 or MdMYB11 improved anthocyanin accumulation in apple calluses, suggesting that MdMYB9 or MdMYB11 acted as an important regulatory factor in anthocyanin biosynthesis [36]. Hu et al. [37] found that MdMYB1 controlled the positively anthocyanin accumulation in apples. MdMYB16 was also an important part of the regulatory network controlling the anthocyanin biosynthetic pathway in apple fruits [38]. The overexpression of MdMYBDL1 enhanced anthocyanin accumulation in apple calli, verifying that MdMYBDL1 functioned as a positive regulator of anthocyanin biosynthesis [39] (Table 1). Wang et al. [40] revealed that overexpressed MdMYB24L resulted in higher anthocyanin contents in the transgenic apple calli than in the wild-type control calli, manifesting that MdMYB24L helped the anthocyanin accumulation of apple as a positive factor. A novel Myb-related sequence, *VlmybA1-3*, was isolated from grape berry [16]. They also further confirmed the gene-regulated anthocyanin biosynthesis in grape berry skin as a positive factor [16]. Similarly, VvMYB5b isolated from grape berry belonged to the R2R3-MYB transcription factor [41]. Overexpression of VvMYB5b in tobacco resulted in the accumulation of anthocyanin, suggesting that the transcriptional mechanisms of VvMYB5b were closely linked to the regulation of the anthocyanin biosynthesis throughout grape berry development [41]. Myb factor, VvMybPA1 and VvMybPA2, promoted anthocyanin biosynthesis in grapevine indicated by Bogs et al. [42] and Terrier et al. [43]. Arabidopsis MYBL2, which encoded an R3-MYB-related protein, positively regulated anthocyanin biosynthesis reported by Dubos et al. [8]; Table 1. Overexpression of MYB112 positively affected the relative expression of key transcription factors of anthocyanin biosynthesis in Arabidopsis, verifying that MYB112 as a positive regulator promoted anthocyanin accumulation [44]. It has been reported that the encoded AN2, an R2R3-MYB transcription factor, was an important regulatory transcription factor in anthocyanin biosynthesis in potato tuber skin by regulating coordinately the expression of multiple anthocyanin biosynthetic genes [29]. Ballester et al. [28] demonstrated that the MYB12 transcription factor played a key role in regulating the anthocyanin biosynthesis in tomato fruit. SlMYB75-OE tomatoes improved anthocyanin content as a key regulator [45]. Yan et al. [46] indicated that SlAN2-like was responsible for anthocyanin biosynthesis in tomato fruits through the CRISPR/Cas9 approach. DEEP PURPLE (DPL) and PURPLE HAZE (PHZ) encoded members of the R2R3-MYB transcription factor family that regulated positively anthocyanin biosynthesis in petunia, and controlled anthocyanin production in vegetative tissues and contributed to floral pigmentation [34]; Table 1. The identified R2R3 MYB transcription factor PavMYB10.1 was involved in the anthocyanin biosynthesis pathway and determined the fruit skin colour in sweet cherry [47]. The isolated R2R3 MYB transcription factor was designated as DcMYB6 from a purple carrot cultivar and overexpression of DcMYB6 in Arabidopsis resulted in enhancing anthocyanin accumulation in both vegetative and reproductive tissues, revealing that DcMYB6 was involved in regulating anthocyanin biosynthesis in purple carrots [48]. Knockout of *DcMYB7* in carrots with purple roots resulted in carrots with yellow roots using the CRISPR/Cas9 system; *DcMYB7* promoted anthocyanin accumulation in carrot roots [49]. Overexpression of LcMYB5 resulted in enhanced biosynthesis of anthocyanins in tobacco and petunia, indicating that LcMYB5 was an R2R3 transcriptional factor which positively regulated anthocyanin biosynthesis [50] (Table 1). Anthocyanin content was increased when R2R3 MYB transcription factor MYB10 or MYB110 was over-expressed, suggesting that kiwifruit anthocyanin biosynthesis was dependent on characterized MYB transcription factors [51]. Over-expressed LrAN2 and LbAN2 induced anthocyanin accumulation in tobacco, indicating that LrAN2 and LbAN2 were closely related to anthocyanin biosynthesis *Lycium ruthenicum* and *Lycium barbarum*, respectively [52]. Overexpression of PsMYB114L and PsMYB12L exhibited significantly higher accumulation of anthocyanins in transgenic Arabidopsis plants, resulting in purple-red leaves. Over-expressed PsMYB114L and PsMYB12L in transgenic apple calli significantly increased the anthocyanins content and led to a change in the callus color to red. These results implied that PsMYB114L and PsMYB12L as positive regulators enhanced anthocyanins accumulation in penoy [53]. Wang et al. [54] indicated that PdMYB118 functions as an essential transcription factor regulating anthocyanin biosynthesis in poplar. The MYB6 overexpressing in transgenic poplar significantly enhanced accumulation of anthocyanin, demonstrating that MYB6 regulated anthocyanin biosynthesis as a positive factor [55]. PaMYB10 overexpression was consistent with the accumulation of anthocyanin in apricot [56]. The isolated RsMYB1, which encoded an R2R3-MYB transcription factor, accumulated high contents of anthocyanins in radish [57] (Table 1). Kim et al. [58] isolated an allele from RsMYB which was named RsMYB1^Short^, and found that RsMYB1^Short^ failed to promote anthocyanin accumulation. CmMYB6 was proposed as a novel activator in Chrysanthemum anthocyanin biosynthesis [59]. GmMYB10 positively regulated anthocyanin biosynthesis in both purple kale and harvested purple kale [60]. LrMYB15 was involved in the positive regulation of anthocyanins in lily by stimulating transcription of anthocyanin biosynthesis genes [61]. LvMYB5-silencing in VIGS experiments significantly reduced anthocyanins accumulation in lily petals [62]. AaMYB2 positively related to anthocyanin biosynthesis in Anthurium spathes [63]. A R2R3 MYB transcription factor named BoPAP1 may play an important role in activating the anthocyanin accumulation in the purple kale [64]. Ectopic expression of EsAN2 in tobacco significantly enhanced the anthocyanin biosynthesis and accumulation, both in leaves and flowers, EsAN2 may regulate positively anthocyanin biosynthesis in *Epimedium sagittatum* [65]. AgMYB2 contained highly conserved R2R3 domain and two anthocyanin characteristic motifs, ANDV motif and KPRPR[S/T]F motif, and revealed that AgMYB2 could enhance anthocyanin biosynthesis and accumulation in celery [66]. The LAP1 MYB transcription factor induced a massive accumulation of anthocyanin pigments and the expression level of *UGT78G1* that increased anthocyanin accumulation was strongly up-regulated by LAP1 in medicago during this process [67]. Anthocyanin production was positively regulated by MYB transcription factor VlmybA2 in tobacco and Arabidopsis [68]. The gene PcMYB10, encoding an R2R3 MYB transcription factor, was involved in the anthocyanin biosynthetic pathway regulation of tobacco and Arabidopsis [69] (Table 1). Transient overexpression of CsMYB33 and CsMYB78 activated anthocyanin biosynthesis in the leaves of *Nicotiana benthamiana* [70]. Over-expressed BoMYB increased the anthocyanidins accumulation in transgenic Arabidopsis, implying that BoMYB positively regulated the synthesis of anthocyanins in kale [71]. RNAi repression of AcMYB123 significantly decreased anthocyanin biosynthesis in kiwifruit [72] (Table 1). The higher expression level of *NsMYB1* may cause higher anthocyanin accumulation in the black fruit in *Nitraria sibirica* Pall, suggesting that NsMYB1 positively induced anthocyanin accumulation [73]. The coloration of purple leaves in *Dendrobium bigibbum* was associated with MYB2, and transient overexpression of MYB2 significantly increased anthocyanin accumulation in tobacco [74] (Table 1).

**Table 1:** Summary of MYB TFs that regulate anthocyanin biosynthesis in plants.

Species	MYB TFs	Effect	References
Apple	MdMYB10	Positive	[21]
Apple	MdMYBA	Positive	[20]
Apple	MdMYB3	Positive	[35]
Apple	MYB110a	Positive	[27]
Apple	MdMYB9 MdMYB11	Positive	[36]
Apple	MdMYB1	Positive	[37]
Apple	MdMYB16	Positive	[38]
Apple	MdMYBDL1	Positive	[39]
Apple	MdMYB24L	Positive	[40]
Grape berry	*VlmybA1-3*	Positive	[16]
Grape berry	VvMYB5b	Positive	[41]
Grapevine	VvMYBPA1 VvMYBPA2	Positive	[42,43]
Arabidopsis	MYBl2	Positive	[8]
Arabidopsis	MYB112	Positive	[44]
Potato	AN2	Positive	[29]
Tomato	MYB12	Positive	[28]
Tomato	SlMYB75	Positive	[45]
Tomato	SlAN2-like	Positive	[46]
Petunia	DPL PHZ	Positive	[34]
Sweet cherry	PavMYB10.1	Positive	[47]
Carrot	DcMYB6	Positive	[48]
Carrot	DcMYB7	Positive	[49]
Petunia	LcMYB5	Positive	[50]
Kiwifruit	MYB10 MYB110	Positive	[51]
Wolfberry	LrAN2 LbAN2	Positive	[52]
Penoy	PsMYB114L PsMYB12L	Positive	[53]
Poplar	PdMYB118	Positive	[54]
Poplar	MYB6	Positive	[55]
Apricot	PaMYB10	Positive	[56]
Radish	RsMYB1	Positive	[57]
Chrysanthemums	CmMYB6	Positive	[59]
Purple kale	GmMYB10	Positive	[60]
Lily	LrMYB15	Positive	[61]
Lily	LcMYB5	Positive	[62]
*Anthurium andraeanu*	AaMYB2	Positive	[63]
Purple kale	BoPAP1	Positive	[64]
*Epimedium sagittatum*	EsAN2	Positive	[65]
Celery	AgMYB2	Positive	[66]
Medicago	LAP1	Positive	[67]
Tobacco Arabidopsis	*VlmybA2*	Positive	[68]
Tobacco Arabidopsis	PcMYB10	Positive	[69]
Mariguana	CsMYB33 CsMYB78	Positive	[70]
Kale	BoMYB	Positive	[71]
Kiwifruit	AcMYB123	Positive	[72]
*Nitraria sibirica* Pall	NsMYB1	Positive	[73]
*Dendrobium bigibbum*	MYB2	Positive	[74]
Arabidopsis	AtMYBL2	Negative	[75]
Arabidopsis	CPC	Negative	[76]
Poplar	MYB182	Negative	[77]
Petunia	MYBx1	Negative	[78]
Tartary buckwheat	FtMYB8	Negative	[79]
Apple	MdMYBL2	Negative	[80]
Chrysanthemum	CmMYB#7	Negative	[81]
Lily	LhR3MYB1 LhR3MYB2	Negative	[82]
Lily	LcMYB1	Negative	[62]
Grapevine	VvMYBC2L2	Negative	[83]
Arabidopsis	MdMYB6	Negative	[84]
Celery	OjMYB1	Negative	[85]
Lettuce	AtMYB60	Negative	[86]
Tobacco	GtMYB1R1 GtMYB1R9	Negative	[87]
Tobacco	AtCPC	Negative	[88]
Gerbera	GhMYB1a	Negative	[89]
Grape hyacinth	MaMYBx	Negative	[90]
Chinese cabbage	BrMYBL2.1	Negative	[91]

### 2.2. MYB TFs Negatively Regulate Anthocyanin Biosynthesis

In Arabidopsis, the identified R3-MYB transcription factor AtMYBL2 acted as a transcriptional repressor and its repressive activity was attributable to the carboxy-terminal region of six amino acids, TLLLFR [75] (Table 1). They also found that AtMYBL2 negatively regulated the biosynthesis of anthocyanin in Arabidopsis [75]. CPC was an R3-MYB transcription factor and found that anthocyanin synthesis genes were significantly downregulated in the 35S:CPC overexpression of Arabidopsis, implying that Arabidopsis was identified as a negative regulator in anthocyanin biosynthesis of Arabidopsis [76]. Overexpression of MYB182 in poplar resulted in reducing anthocyanin levels, indicating that MYB182 as a negative regulator inhibited anthocyanin biosynthesis [77] (Table 1). MYBx1 can negatively regulate anthocyanin production and influence floral pigmentation by repressing the activity of the MBW complex in petunia [78]. Overexpression of FtMYB8 reduced the accumulation of anthocyanin in Arabidopsis, implying that FtMYB8 negatively regulated anthocyanin biosynthesis in Tartary buckwheat [79]. The MdMYBL2-overexpressing callus exhibited lower anthocyanin contents than of the control, suggesting that MdMYBL2 functioned as a negative regulator of anthocyanin biosynthesis in apple [80]. Xiang et al. [81] illustrated that CmMYB#7, an R3 MYB transcription factor, was a negative regulator of anthocyanin biosynthesis in chrysanthemum. Two R3-MYBs, LhR3MYB1 and LhR3MYB2, which were identified and found that they had a C2 suppressor motif downstream of a single MYB repeat. Meanwhile, both stable and transient overexpressed LhR3MYB1 and LhR3MYB2 showed inhibition of anthocyanin biosynthesis in tobacco plants. Thus, LhR3MYB1 and LhR3MYB2 suppressed anthocyanin biosynthesis in lily as a passive factor [82] (Table 1). LvMYB1, an R2R3-MYB transcription factor, inhibited anthocyanin biosynthesis in lily flowers [62]. The isolated VvMYBC2L2 from Rose, an R2R3-MYB transcription factor, was involved in the regulation of anthocyanin biosynthesis in grapevine as a transcriptional repressor [83]. MdMYB6 over-expression inhibited the activities of an anthocyanin biosynthesis-related enzyme and the expression levels of some bHLH genes, indicating that the MdMYB6 transcription factor is an important repressor of anthocyanin biosynthesis in Arabidopsis [84] (Table 1). OjMYB1, an encoding R2R3-MYB transcription factor, could enhance the anthocyanins content in Arabidopsis [85]. The identified AtMYB60 suppressed anthocyanin biosynthesis in the lettuce plant [86]. GtMYB1R1 and GtMYB1R9 acted as antagonistic transcription factors of anthocyanin biosynthesis in gentian flowers [87] (Table 1). MYB-type transcription factor AtCPC acted as a repressor of anthocyanin production [88] (Table 1). In gerbera and tobacco, overexpressed *GhMYB1a* significantly decreased anthocyanin content [89]. MaMYBx, an R3 MYB transcription factor, acted as a repressor of anthocyanin accumulation in grape hyacinth [90]. R3 MYB transcription factor BrMYBL2.1 suppressed anthocyanin biosynthesis in Chinese cabbage by blocking MBW complex activity [91].

As mentioned above, many MYB TFs positively regulated anthocyanin biosynthesis in plants including Apple, Grape berry, Arabidopsis, Potato, Tomato, etc, which belong to R2R3 and R3 MYB TFs. MYB TFs that negatively regulated anthocyanin synthesis in plants including Arabidopsis, Poplar, Apple, and so on have also been identified. They usually belong to R3 MYB TFs, but some of them belong to R2R3 MYB TFs. To explore the roles of MYB TFs in plants, some methods are implemented, such as overexpression, RNAi, CRISPR/Cas9, and VIGS. So far, it has been found that the MYB TFs regulating apple anthocyanins accumulation was more than those regulating other plants. Further research is required to investigate the role of MYB TFs in regulating anthocyanin biosynthesis in more plants.

## 3. The Signaling Pathway Regulated by MYB TFs during Anthocyanin Biosynthesis

### 3.1. Jasmonic Acid (JA) Signaling Pathway

**Figure 1:**
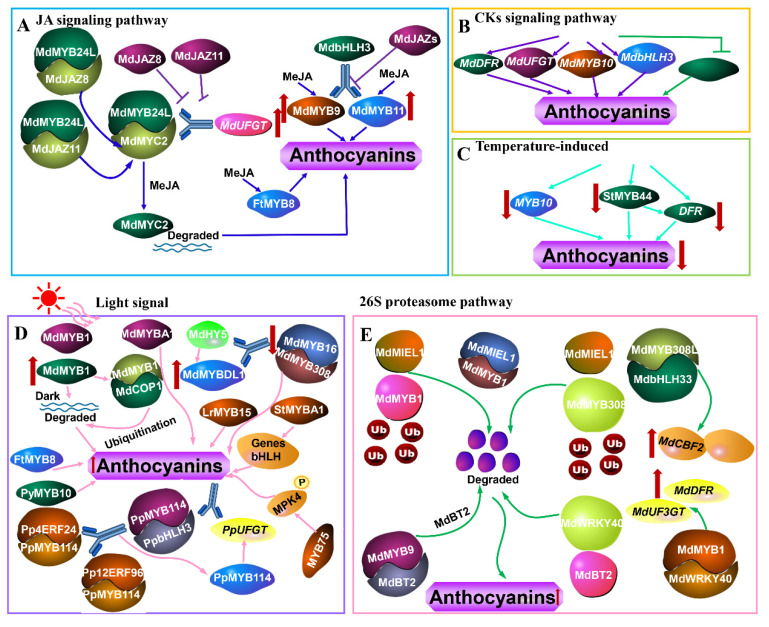
The signaling pathway medicated by MYB TFs during anthocyanin biosynthesis, such as JA signaling pathway (**A**), cytokinins (CKs) signaling pathway (**B**), temperature-induced (**C**), light signal (**D**), 26S proteasome pathway (**E**). JA, jasmonic acid; MeJA, jasmonic acid methyl ester.

The encoded nuclear protein, MdMYB24L, interacted with JA signaling factors including MdJAZ8, MdJAZ11, and MdMYC2 that were observed [40] (Figure 1A). The MdJAZ8 and MdJAZ11 directly targeted MdMYC2 protein, which are rapidly degraded in the jasmonic acid methyl ester (MeJA) treatment. The interaction between MdMYB24L and MdMYC2 further increased the transcriptional level of *MdUFGT*; nevertheless, this effect was weakened by MdJAZ8 and MdJAZ11 [40]. Overexpression of MdMYB9 or MdMYB11 promoted anthocyanin accumulation in apple calluses, and the accumulation was further enhanced by MeJA. MdbHLH3 was recruited to the promoters of *MdMYB9* and *MdMYB11* and regulated their transcription, but MdJAZs inhibited the recruitment of MdbHLH3 to the promoters of *MdMYB9* and *MdMYB11* [36] (Figure 1A). The expression of *FtMYB8* was markedly induced by JA during anthocyanin biosynthesis [79] (Figure 1A).

### 3.2. Cytokinins (CKs) Signaling Pathway

CKs are one of the major plant hormones that is widely distributed in various tissues of plants [92]. There are two types of CKs: adenine-type CKs represented by kinetin, zeatin, and 6-benzylaminopurine (6-BA), and phenylurea-type CKs such as diphenylurea and thidiazuron (TDZ) [92]. It was well known that CKs induced anthocyanin biosynthesis, and anthocyanin is also regulated by MYB transcription factors [80,92]. CKs treatments (6-BA) upregulated the relative expression of *MdDFR*, *MdUFGT*, *MdMYB10,* and *MdbHLH3* genes [80] (Figure 1B). Wang et al. [80] also observed that MdMYBL2 was a negative factor in anthocyanin biosynthesis and was inhibited by CKs treatments, suggesting that MdMYBL2 influenced the CKs-regulated anthocyanin biosynthesis in red-fleshed apples.

### 3.3. Temperature-Induced

Lin-wang et al. [32] showed that the high temperature significantly down-regulated the expression of *MYB10,* which was responsible for reductions in anthocyanin accumulation. StMYB44 inhibited anthocyanin accumulation as a negative regulator by suppressing the activity of the *DFR* promoter [93] (Figure 1C). The high temperature reduced anthocyanin accumulation in potato flesh by enhancing the expression of StMYB44 and down-regulating the activation of the *DFR* promoter activity [93].

### 3.4. Light Signal

MdMYB1 and MdMYBA were important regulators of light-induced anthocyanin biosynthesis in apple fruits [19,20]. Under the light condition, MdMYB1 was accumulated, whereas it was degraded via a ubiquitin-dependent pathway in the dark. MdMYB1 interacted with nuclear MdCOP1proteins and MdCOP1 proteins were responsive to the ubiquitination and degradation of MdMYB1 protein in the dark, and they were involved in the light-controlled stability of the MdMYB1 protein [94] (Figure 1D). Liu et al. [39] indicated that MdHY5 bound to the G-box element of the MdMYBDL1 promoter to activate its expression. MdMYB16 can form a dimer with MdMYB308, which negatively regulated anthocyanin biosynthesis. Furthermore, MdMYBDL1 and MdHY5 suppressed the promoter activities of MdMYB16 and MdMYB308. These results implied that MdHY5 responded to light signals and was upstream of various kinds of MYB transcription factors in the regulation of anthocyanin accumulation in apples [39]. LrMYB15 transcription ceased completely when plants were kept in shaded conditions and the colors of the flower buds faded, indicating the regulated light-induced anthocyanin accumulation [61]. Applying the dark conditions to the tobacco plants overexpressing StMYBA1 transiently can down-regulate the expression levels of biosynthetic pathway genes and bHLH transcription factors, and subsequently reduced anthocyanin accumulation. The results suggested that StMYBA1 can positively regulate anthocyanin biosynthesis in tobacco, and light was required for its function on anthocyanin accumulation [95] (Figure 1D). PyMYB10 had core sequences of *cis*-acting regulatory elements involved in light responsiveness and showed that the expression of *PyMYB10* was induced by light during anthocyanin biosynthesis [96]. Dark treatment repressed the expression of FtMYB8 during anthocyanin accumulation [79]. The transcript levels of *PaMYB10, PaPAL*, *PaCHS*, *PaCHI*, *PaF3H*, *PaDFR*, *PaLDOX,* and *PaUFGT* were inhibited by bagging treatment, resulting in the decrease in anthocyanin contents [56]. Interaction of Pp4ERF24 and Pp12ERF96 with PpMYB114 regulated light-induced anthocyanin biosynthesis by promoting the interaction between PpMYB114 and PpbHLH3 and enhancing the expression of PpMYB114-induced *PpUFGT* [97]. Li et al. [98] revealed that MPK4 phosphorylation of MYB75 increased its stability and was essential for light-induced anthocyanin accumulation.

### 3.5. 26S Proteasome Pathway

MdMIEL1 functioned as a ubiquitin 3 ligase to ubiquitinate MdMYB1 protein, followed by degradation through a 26S proteasome pathway, demonstrated that MdMIEL1 negatively regulated anthocyanin accumulation [99]. The interaction between MdMIEL1 and MdMYB1 proteins was verified during this process [99] (Figure 1E). MdMYB308L as a positive regulator of anthocyanin biosynthesis interacted with MdbHLH33 and enhanced its binding to the promoters of *MdCBF2* and *MdDFR*. However, MdMIEL1 as a negative regulator was identified to be a MdMYB308L-interacting protein and promoted the ubiquitination degradation of MdMYB308L via a 26S proteasome pathway in the apple [100]. MdBT2 interacted with MdMYB9 and negatively regulated the abundance of MdMYB9 protein through the 26S proteasome pathway. The degradation of MdMYB9 by MdBT2 down-regulated the expression levels of MdMYB9-mediated anthocyanin-related genes and reduced the accumulation of anthocyanin, which functioned in an MdCUL3-independent pathway [101]. MdMYB1, a positive regulator of anthocyanin biosynthesis, interacted with MdWRKY40 and enhanced its binding to the promoters of *MdDFR* and *MdUF3G*T. However, MdBT2 interacted with MdWRKY40 and degraded its protein abundance by the 26S proteasome pathway [102] (Figure 1E).

### 3.6. NAC TFs

LcR1MYB1, as one R1-MYB type MYB, was identified to physically interact with LcNAC13 and reversed the effect of LcNAC13. LcNAC13 could bound anthocyanin structural genes *LcCHS1/2*, *LcCHI*, *LcF3H*, *LcF3′H*, *LcDFR*, and *LcMYB1* and negatively regulated the expression of these genes. To sum up, LcNAC13 and LcR1MYB1 may act together to antagonistically regulate anthocyanin biosynthesis during litchi fruit ripening, which helps to provide new insights into the regulatory networks of anthocyanin biosynthesis [103].

### 3.7. bHLH TFs

Endogenous bHLH partners can be stimulated by overexpressing exogenous gene StMYBA1 in tobacco, and the elevated expression levels of bHLH partners were essential for anthocyanin production in plant tissues [95]. LcbHLH1 and LcbHLH3 were essential partners of LcMYB1 in regulating the anthocyanin production in tobacco and also probably in litchi [104].

## 4. Regulatory Mechanisms of MYB TFs in Anthocyanin Biosynthesis

### 4.1. Modulation of Gene Expression by MYB TFs

In transgenic tobaccos, overpressed-MdMYB3 had resulted in transcriptional activation of *CHS*, *CHI*, *UFGT*, and *FLS* genes, which exhibited increased anthocyanins accumulation in flowers [35] (Table 2). The expression levels of *OsPAL*, *CHS*, *ANS*, and *MYB55* genes were up-regulated in the *purple leaf* (*pl*) mutant compared to the wild type, which showed the enhancement of anthocyanins contents in leaves of rice [105]. VvMYBC2L2 as a nuclear protein could remarkably down-regulate structural genes including *CHS*, *DFR*, *LAR* and *UFGT* and regulatory genes including *AN1a* and *AN1*b, resulting in the increase in anthocyanin content [83]. The expression of *PyMYB10* in the pear skin was positively correlated with *CHS*, *PAL*, *CHI*, *DFR*, *ANS,* and *F3H* genes [96]. The expression levels of anthocyanin biosynthetic including *CHS*, *CHI*, *F3H*, *F3′H*, *DFR*, *LDOX,* and *UGT78D2* in the transgenic Arabidopsis carrying AgMYB2 were significantly up-regulated [66] (Table 2). Feng et al. [85] found that OjMYB1 could up-regulate the expression levels of the structural genes-related anthocyanins biosynthesis including *CHS*, *CHI*, *F3H*, *F3′H*, *DFR*, *LDOX,* and *UGT78D2* in Arabidopsis to enhance the anthocyanins biosynthesis. Overexpressing EsAN2 significantly upregulated the relative expression of *CHS*, *CHI*, *F3H*, *F3′H*, *FLS*, *DFR*, *ANS*, *An1a,* and *An1b* genes in tobacco [65]. RsMYB1 could up-regulate the relative expression of anthocyanin biosynthetic-related genes *F3H*, *DFR*, and *ANS* involved in anthocyanin production [57]. AaMYB2 appeared to regulate the expression of *F3H*, *ANS*, *CHS* genes and is possibly involved in anthocyanins biosynthesis [98]. Wang et al. [40] reported that the expression levels of the anthocyanin biosynthesis structural genes *UFGT* and *DFR* were up-regulated in the transgenic calli of over-pressed MdMYB24L (Table 2). The expression levels of *DFR*, *UFGT*, *MYB10,* and *bHLH3* genes were strongly suppressed in the MdMYBL2-overexpressing callus, suggesting that MdMYBL2 negatively regulated anthocyanin accumulation in apple [80]. PsMYB114L and PsMYB12L both enhance anthocyanin accumulation by down-regulating the relative expression of *FLS* and *ANR* genes and up-regulating the relative expression of *DFR* and *ANS* genes [53]. *DcMYB7* could activate expression of bHLH3 and structural genes in the anthocyanin biosynthetic pathway including *CHS1*, *CHI1*, *F3H1*, *F3′H1*, *FR1*, *LDOX1*, and *UCGalT1* [49]. Overexpression of DcMYB6 in Arabidopsis upregulated the transcriptional level of *CHS1*, *CHI1*, *F3H1*, *F3′H1*, *DFR*, *LDOX1*, and *UGT78D2* genes [48] (Table 2). Expression of *CHS1*, *CHI1*, *F3H*, *F3′H*, *F3′5′H*, *DFR2,* and *ANS1* was improved in poplar during the transient over-expressed expression of PdMYB118 [54]. NsMYB1 could promote the transcript of *PAL*, *C4H*, *4CL*, *F3H*, *F3′H*, *DFR,* and *ANS* of black fruit in *Nitraria sibirica* Pall, and induce the anthocyanin accumulation in tobacco [73]. LvMYB5-silencing significantly up-regulated the expression levels of *CHS*, *DFR*, and *ANS* genes [62] (Table 2). Anthocyanin biosynthesis genes *DFR*, *ANS,* and *CHS* were significantly up-regulated in the transient MYB2-overexpressing [74]. Transient overexpression of GhMYB1a resulted in the increase in *MYB1*, *CHS*, *F3H*, *F3′H*, *DFR*, *ANS*, *FLS*, *UFGT*, *MYB10,* and *MYC1* genes [89]. SlAN2 acted as an activator of the expression of structural genes including *PAL*, *C4H*, *4CL*, *CHS1*, *CHS2*, *CHI*, *CHI-like*, *F3H*, *F3′5′H*, *DFR*, *ANS*, *3GT*, *RT*, *AAC*, *PAT*, *GST,* and regulator genes including *AN1*, *AN11*, *MYBATV*, *MYBATV-like*, *TRY*, *MYB76,* and positively regulated the anthocyanins accumulation [46]. Overexpression of MaMYBx significantly repressed the relative expression of *C4H*, *4CL*, *F3′5′H*, *FLS*, *DFR*, *ANS*, *AN2*, *AN1a*, and *AN1b* in tobacco [90] (Table 2).

### 4.2. DNA Binding of MYB TFs

MdMYBA bound specifically to the *MdANS* promoter region, which increased anthocyanin accumulation in the apple [20] (Table 3). AtMYBL2 bound directly to the TT8 protein, and this complex suppressed the expression of *DFR* and *TT8* during anthocyanin pathway [75] (Table 3). MdMYB24L positively regulated anthocyanin biosynthesis via directly binding to the MYB-binding site motifs in the promoters of *MdDFR* and *MdUFGT* and activating its transcriptional [40]. SlMYB75 as a positive regulator of anthocyanin biosynthesis was able to directly bind to the MYBPLANT and MYBPZM *cis*-regulatory elements and to activate the promoters of the *LOXC*, *AADC2,* and *TPS* genes [45] (Table 3). CmMYB#7 competed with CmMYB6, which together with CmbHLH2 was an essential component of the anthocyanin activation complex, for interaction with CmbHLH2 through the bHLH binding site in the R3 MYB domain. This reduced binding of the CmMYB6–CmbHLH2 complex inhibited its ability to activate *CmDFR* and *CmUFGT* promoters [81]. DcMYB7 could activate directly *DcUCGXT1* and *DcSAT1* to regulate the glycosylation and acylation of anthocyanins [48]. MdMYB16 and LESMdMYB16 interacted the promoters of *MdANS* and *MdUFGT* yeast, respectively [38] (Table 3). PdMYB118 can directly activate the promoters of *CHS1*, *DRF2,* and *ANS1* genes which functioned as an essential transcription factor regulating anthocyanin biosynthesis in poplar [54] (Table 3). AcMYB123 and AcbHLH42 were involved in the regulation of anthocyanin biosynthesis by activating promoters of *AcANS* and *AcF3GT1* genes [72]. Nakatsuka et al. [106] reported that co-expression of GtMYB3 and GtbHLH1 in transient expression assay could enhance the promoter activities of anthocyanin biosynthetic genes including *CHS*, *F3′5′H,* and *5AT* in tobacco BY2 cells. *GtDFR* promoter was activated by the GtMYB3-GtbHLH1 complex [87] (Table 3). MYBA1, MYBA6.1, and MYBA7 promoted anthocyanin accumulation in grapevine hairy roots by activating the promoters of *UFGT* and *3AT* but only MYBA1 inducing *F3′5′H* promoter [107]. PavMYB10.1 bound to the promoter of *PavANS* and *PavUFGT*, which was involved in the regulation of anthocyanin accumulation [47] (Table 3). LcMYB5 enhanced anthocyanin biosynthesis by directly activating the expression of *DFR* or by indirectly up-regulating the expressions of *bHLH1* [50] (Table 3). CmMYB6 significantly activated the *CmDFR* promoter. The activity of CmDFR was further enhanced by the combination of CmMYB6 and MrbHLH1, leading to anthocyanin accumulation [59] (Table 3). LvMYB1 bound to the promoter of the *LvANS* gene, and enhanced its expression, thereby promoting anthocyanin synthesis [62]. GhMYB1 significantly activated the promoters of *NtCHS* and *NtFLS* genes, which are required for anthocyanin biosynthesis in gerbera [89]. MaMYBx bound *MaMybA* and *MabHLH1* promoters and suppressed their expression, thereby reducing anthocyanin accumulation in grape hyacinth [90].

### 4.3. Interactions between MYB TFs and Other Proteins

**Figure 2:**
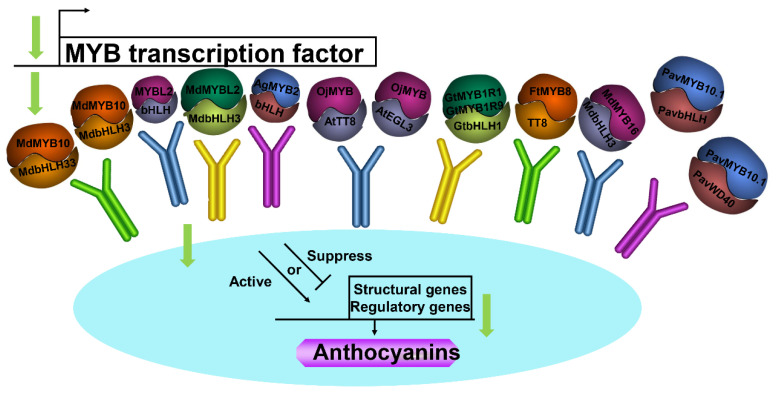
Schematic model of the interaction between MYB TFs with other proteins. TFs, transcription factors.

Efficient induction of anthocyanin biosynthesis in transient assays by MdMYB10 was dependent on the co-expression of two distinct bHLH proteins from the apple: MdbHLH3 and MdbHLH33 [21] (Figure 2). MYBL2 interacted with bHLH proteins and modulated the expression of anthocyanin biosynthesis-related genes, which influenced anthocyanin accumulation [8]. Wang et al. [80] indicated that MdMYBL2 interacted with MdbHLH3 in the apple and the interaction enhanced anthocyanin accumulation by regulating the expression of anthocyanin biosynthesis-related genes (Figure 2). Yeast two-hybrid assay confirmed that EsAN2 was capable of interacting with NtAn1a, NtAn1b, EsTT8, and AtTT8 regulators of the anthocyanin biosynthetic pathway [65] (Figure 2). The interaction between AgMYB2 and bHLH proteins was shown by yeast two-hybrid assay [66]. OjMYB1 could interact with AtTT8 and AtEGL3 proteins in yeast [85]. Nakatsuka et al. [87] demonstrated that both GtMYB1R1 and GtMYB1R9 proteins interacted with the GtbHLH1 protein, previously identified as an anthocyanin biosynthesis regulator in gentian flowers. FtMYB8 inhibited anthocyanin accumulation by inhibiting *TT12* expression and interacting with TT8 [79]. MdMYB16 interacted with MdbHLH3, which may control the anthocyanin biosynthetic pathway [38]. PavMYB10.1 interacted with proteins PavbHLH and PavWD40 using yeast two-hybrid assays and chromatin immunoprecipitation assays during anthocyanin biosynthesis [47] (Figure 2).

## 5. Conclusions and Future Perspective

In conclusion, in plants, some MYB TFs could regulate, positively or negatively, anthocyanins accumulation. JA signaling pathway, CK signaling pathway, temperature-induced, light signal, 26S proteasome pathway, NAC TFs, and bHLH TFs are essential pathways for regulating the anthocyanin biosynthesis of plants. Additionally, we have also concluded that MYB TFs induced the expression of structural genes and regulator genes, bound and activated or suppressed anthocyanin biosynthesis-related target genes, and interacted with other proteins, which improved or inhibited anthocyanins accumulation in plants. These insights might be advantageous in identifying new varieties and breeding new cultivars.

Nowadays, although it is a well-known fact that MYB TFs-medicated signaling pathways regulate anthocyanins accumulation, the signaling pathways induced by MYB TFs involved in this process still needs to be further elucidated. Additionally, MYB TFs regulated structural genes and regulator genes, bound and activated or suppressed target genes, and interacted with other proteins during anthocyanin biosynthesis. Clearly, protein modifications induced by MYB TFs including *S*-nitrosylation, methylation, phosphorylation, and so on are lacking. More research works will improve knowledge concerning the regulator mechanism of MYB TFs in the anthocyanin biosynthesis pathway with the aim of identifying new varieties and breeding new cultivars.

## Figures and Tables

**Table 2:** The genes regulated by MYB TFs during anthocyanin biosynthesis.

MYB TFs	Genes	Effect	References
MdMYB3	*CHS CHI UFGT FLS*	Activate	[35]
OsPL	*PAL CHS ANS MYB55*	Suppress	[105]
VvMYBC2L2	*CHS DFR LAR UFGT AN1a AN1b*	Suppress	[83]
PyMYB10	*CHS PAL CHI DFR ANS F3H*	Activate	[96]
AgMYB2	*CHS CHI F3H F3′H DFR LDOX UGT78D2*	Activate	[66]
OjMYB1	*CHS CHI F3H F3′H DFR LDOX UGT78D2*	Activate	[85]
EsAN2	*CHS CHI F3H F3′H FLS DFR ANS An1a An1b*	Activate	[65]
RsMYB1	*F3H DFR ANS*	Activate	[57]
AaMYB2	*F3H ANS CHS*	Activate	[98]
MdMYB24L	*UFGT DFR*	Activate	[40]
MdMYBL2	*DFR UFGT MYB10 bHLH3*	Suppress	[80]
PsMYB114L PsMYB12L	*FLS ANR*	Suppress	[53]
PsMYB114L PsMYB12L	*DFR ANS*	Activate	[53]
DcMYB7	*CHS1 CHI1 F3H1 F3′H1 FR1 LDOX1 UCGalT1*	Activate	[49]
DcMYB6	*CHS1 CHI1 F3H1 F3′H1 DFR LDOX1 UGT78D2*	Activate	[48]
PdMYB118	*CHS1 CHI1 F3H F3′H F3′5′H DFR2 ANS1*	Activate	[54]
NsMYB1	*PAL C4H 4CL F3H F3′H DFR ANA*	Activate	[73]
LvMYB5	*CHS DFR ANS*	Activate	[62]
MYB2	*DFR ANS CHS*	Activate	[74]
GhMYB1a	*MYB1 CHS F3H F3′H DFR ANS FLS UFGT MYB10 MYC1*	Activate	[89]
SlAN2	*PAL C4H 4CL CHS1 CHS2 CHI CHI-like F3H F3′5′H DFR ANS 3GT RT AAC PAT GST AN1 AN11 MYBATV MYBATV-like TRY MYB76*	Activate	[46]
MaMYBx	*C4H 4CL F3′5′H FLS DFR ANS AN2 AN1a AN1b*	Suppress	[90]

**Table 3:** The target genes bound by MYB TFs during anthocyanin biosynthesis.

Species	MYB TFs	Target Genes	References
Apple	MdMYBA	*MdANS*	[20]
Arabidopsis	AtMYBL2	*DFR TT8*	[75]
Apple	MdMYB24L	*MdDFR MdUFGT*	[40]
Tomato	SlMYB75	*LOXC AADC2 TPS*	[45]
Chrysanthemum	CmMYB6	*CmDFR CmUFGT*	[81]
Carrot	DcMYB7	*DcUCGXT1 DcSAT1*	[49]
Poplar	PdMYB118	*CHS1 DRF2 ANS1*	[54]
Kiwifruit	AcMYB123	*AcANS AcF3GT1*	[72]
Tobacco	GtMYB3	*CHS F3′5′H 5AT*	[106]
Tobacco	GtMYB3-GtbHLH1	*GtDFR*	[87]
Grapevine	MYBA1, MYBA6.1 MYBA7	*UFGT 3AT*	[107]
Grapevine	MYBA1	*F3′5′H*	[107]
Sweet cherry	PavMYB10.1	*PavANS PavUFGT*	[47]
Litchi	LcMYB5	*Bhlh1*	[50]
Chrysanthemum	CmMYB6	*CmDFR*	[59]
Lily	LvMYB1	*LvANS*	[62]
Gerbera	GhMYB1	*NtCHS NtFLS*	[89]
Grape hyacinth	MaMYBx	*MaMybA MabHLH1*	[90]

## Data Availability

Not applicable.
